# Plant and mouse EB1 proteins have opposite intrinsic properties on the dynamic instability of microtubules

**DOI:** 10.1186/s13104-020-05139-6

**Published:** 2020-06-22

**Authors:** Arthur T. Molines, Virginie Stoppin-Mellet, Isabelle Arnal, Frédéric M. Coquelle

**Affiliations:** 1 Department of Cell Biology, Institute for Integrative Biology of the Cell (I2BC), CEA, CNRS, Université Paris-Saclay, 91198 Gif-Sur-Yvette Cedex, France; 2 Université Grenoble Alpes, Grenoble Institut des Neurosciences, BP170, 38042 Grenoble Cedex 9, France; 3 Present Address: Department of Cell and Tissue Biology, University of California San Francisco, San Francisco, CA 94143 USA; 4 Present Address: Institut Curie–Centre de Recherche, CNRS, UMR3347/INSERM U1021, Université Paris-Saclay, 91405 Orsay, France

**Keywords:** Microtubules, End-binding protein 1 (EB1), Cytoskeleton, Microtubule dynamic instability, Plants

## Abstract

**Objective:**

Most eukaryotic cells contain microtubule filaments, which play central roles in intra-cellular organization. However, microtubule networks have a wide variety of architectures from one cell type and organism to another. Nonetheless, the sequences of tubulins, of Microtubule Associated proteins (MAPs) and the structure of microtubules are usually well conserved throughout the evolution. MAPs being known to be responsible for regulating microtubule organization and dynamics, this raises the question of the conservation of their intrinsic properties. Indeed, knowing how the intrinsic properties of individual MAPs differ between organisms might enlighten our understanding of how distinct microtubule networks are built. End-Binding protein 1 (EB1), first described as a MAP in yeast, is conserved in plants and mammals. The intrinsic properties of the mammalian and the yeast EB1 proteins have been well described in the literature but, to our knowledge, the intrinsic properties of EB1 from plant and mammals have not been compared thus far.

**Results:**

Here, using an in vitro assay, we discovered that plant and mammalian EB1 purified proteins have different intrinsic properties on microtubule dynamics. Indeed, the mammalian EB1 protein increases microtubules dynamic while the plant EB1 protein stabilizes them.

## Introduction

The regulation of the intra-cellular network of cytoskeletal filaments is a critical part of cellular function in eukaryotes. Microtubules are found in most eukaryotes and their structure is well preserved [1]. Depending on the kingdom, microtubules are involved in different processes [2]. Nonetheless, Microtubule Associated Protein (MAP), which are major regulators of microtubule dynamics, are present across these organisms. One might assume that protein properties are conserved between organisms, but such an assumption is worth testing.

Indeed, the End-Binding protein 1 (EB1), known to be a major organizer of the complex system of proteins recruited at the growing tip of microtubules in mammalian cells [3], is conserved in plants [4] and yeasts [5] (Mal3p in fission yeast, Bim1p in budding yeast). In mammalian cells and yeasts, EB1 recruits partners through its EBH (End Binding Homology) domain or its EEY sequence in C-terminal domain. The EBH domain allows EB1 to recruits proteins containing S(X)IP domains, while the EEY sequence found in the C-terminus allows EB1 to interact with proteins containing CAP-Gly domains, such as P150^GLUED^ and CLIP-170 [6, 7].

**Fig. 1:**
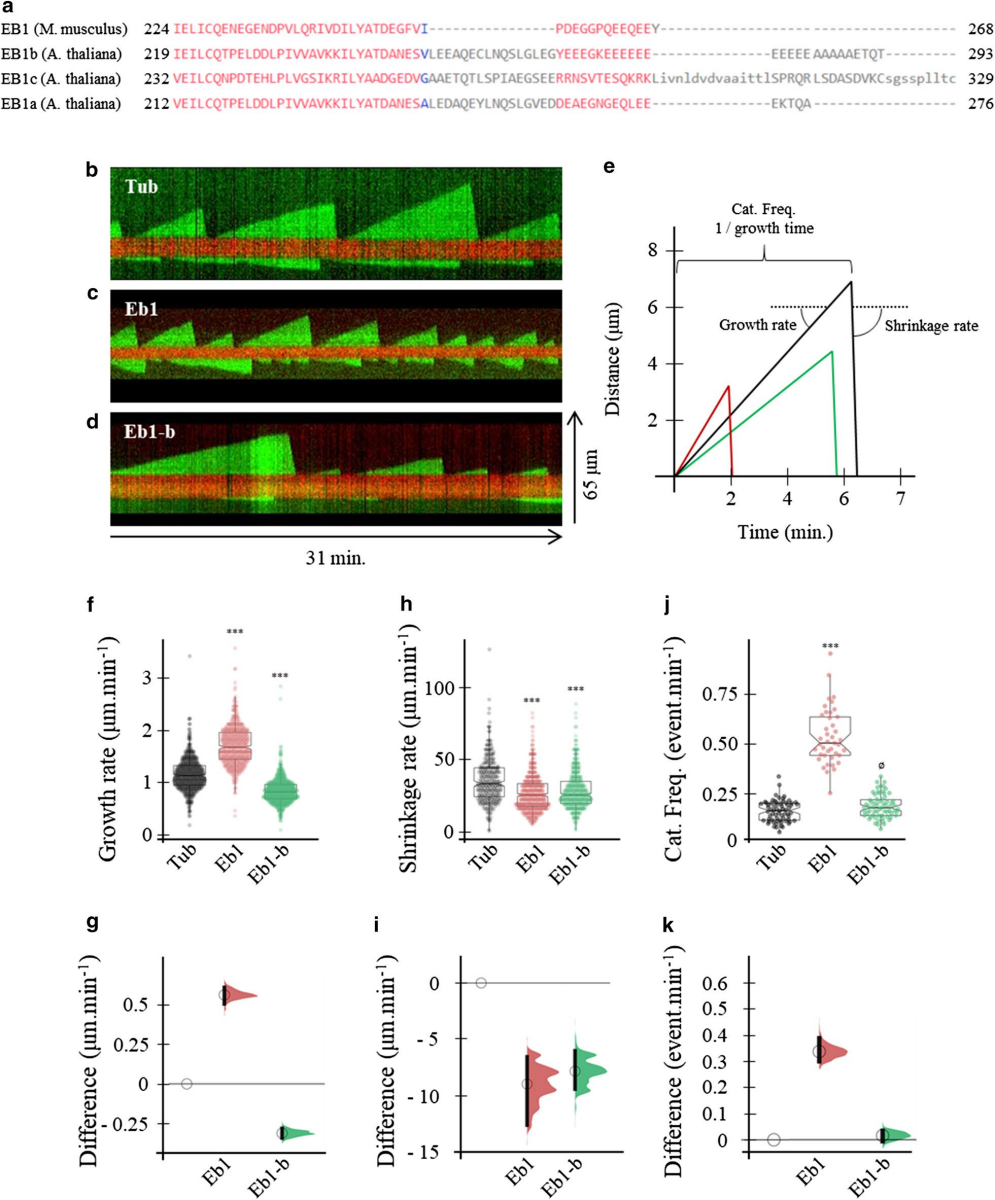
Comparison of the effect of mammalian and plant EB1 protein on microtubule dynamics. **a** Protein sequence homology between EB1 proteins from mice (*Mus musculus*) and plant (*Arabidopsis thaliana*). Mice EB1 (Q61166.3), EB1a (Q7XJ60.1) EB1b (Q9FJJ5.1) EB1c (Q9FGQ6.1). Note that the final EEY motif found in the EB1 proteins from mice (and other mammals) is not present in the plant EB1. Representative kymographs of microtubule seeds (in red) growing in presence of 15 μM of tubulin (**b)**, 15 μM of tubulin and 75 nM of EB1 from mammals **(c)**, and 15 μM of tubulin and 75 nM of EB1-b from plant **(d)**. All kymographs are oriented with the + end to the top. **e** is a schematic of the ideal microtubule tracks for each condition based on the average parameters of their dynamic instability estimated from the kymographs in **(b–d)**. The schematic also shows how the parameters are estimated from the kymographs. Graphs in **(f–j)** show the growth rate **(f)**, the shrinkage rate **(h)** and the catastrophe frequency **(j)** of microtubules grown in 15 μM of tubulin (black), 15 μM of tubulin and 75 nM of EB1 from mammals (red) or 15 μM of tubulin 75 nM of EB1-b from plant (green) as jittered dots (visibility: 0.1). The summary of the data is shown as a boxplot, with the box indicating the interquartile range (IQR), the whiskers showing the range of values that are within 1.5*IQR and a horizontal line indicating the median (visibility: 0.9). The notches represent for each median the 95% confidence interval (approximated by 1.58*IQR/sqrt(n)). Asterisks indicates statistically significant difference, (p < 0.001) obtained from the bootstrap analysis (see methods for details). Plots in **(g, i, k)** show the absolute effect size, relative to the 15 μM tubulin condition for the growth rate **(g)**, the shrinkage rate **(i)** and the catastrophe frequency **(k)**. The bootstrap samples that are used to calculate the 95% confidence interval of the effect size are shown as a distribution. 95% confidence intervals are represented as black bars

Arabidopsis possesses three orthologs of EB1 [4, 8, 9], AtEB1-a, AtEB1-b and AtEB1-c. AtEB1-a and AtEB1-b share around 60% of similarity at the amino-acid level with EB1 from mammals while AtEB1-c is more divergent. The EBH domain is present in EB1 from plants and S(X) IP domains are found in numerous proteins. Interestingly, there is no known CAP-Gly containing proteins in *Arabidopsis* and the plants orthologs of EB1 lack the EEY domain (Fig. 1a) but possess instead a stretch of acidic amino-acids [4].

On one hand, the conservation of EB1 localization at the tip of growing microtubules between mammals and plants and the relatively high similarity of sequence suggest a conservation of its properties. On the other hand, since the separation of the viridiplantae and the opisthokonts clades, EB1 intrinsic properties could have diverged between plants and mammals/fungi. The mammalian version of EB1 has been purified and its effect on microtubule dynamics extensively studied both in vivo and in vitro [10]. In contrast, the plant EB1 roles in development and microtubule network organization have been explored using knock-out mutants and loss-of-function mutants, revealing functions in touch sensing and microtubule bundling [11–13] but its intrinsic properties have not been extensively studied in vitro yet.

We set out to evaluate the effect of purified plant and mammalian EB1 proteins on microtubule dynamics in an in vitro reconstituted system. We compared the proteins in identical conditions (same tubulin, tubulin concentration and concentration of EB1) by measuring their effect on the parameters of microtubule dynamic instability (rates of growth and shrinkage, frequency of catastrophe).

## Main text

### Methods

#### Protein expression and purification

See supplemental material for the methods on proteins purification Additional file 1.

#### Sample preparation

See supplemental material for details on sample preparation.

#### TIRF microscopy

Samples were observed on an inverted microscope (Nikon, TI-E) with a 60X 1.49 TIRF immersion oil objective. The microscope was equipped with iLAS^2^ TIRF system (Roper Scientific), a Evolve 512 camera (EMCCD, Photometrics), and controlled with Metamorph (Molecular Devices). Observations were done with two excitation lasers at 491 nm and 561 nm.

#### Estimation of dynamic instability parameters from the movies

Kymographs were obtained from the calibrated movies using KymoToolbox ImageJ plugin (F. Cordelières). Rates of growth and shrinkage were measured for each event and then pooled. The catastrophe frequency was estimated per microtubule then pooled per condition.

#### Statistical analysis

For the control, the mammalian EB1 and the plant EB1 conditions 66, 41, and 65 MT tracks were analyzed respectively. The data come from at least 2 independent experiments. These allowed the observation of 533, 533, and 652 events of growth and 277, 515, and 462 events of catastrophe respectively. For each dataset, we choose to mention the median and the 95% confidence interval in the text because the data are not distributed normally. The distributions were compared using the PlotOfDifferences web-app (BioRxiv, Goedhart, 10.1101/578575). p-values and effect size were obtained from the bootstrap analysis available in the web-app. For more information about effect size please go to the following website: https://thenode.biologists.com/quantification-of-differences-as-alternative-for-p-values/research/.

## Results

We used TIRF microscopy to compare the effect of 75 nM of EB1 from plant or mice on microtubule (MT) polymerized from 15 μM of tubulin. Samples were prepared at room temperature in micro-chambers and rapidly mounted on the microscope at 36 °C to be observed at 0.2 fps for at least 30 min (see Methods for more details).

MTs dynamic instability parameters were determined from manual kymograph analysis (see Methods for more details) (Fig. 1a–d). In the control condition (Tub), in absence of any EB1 protein, MTs polymerization rate is 1.1 [1.08–1.15] μm min^−1^ (Median [95% confidence interval]), the depolymerization rate is 30 times higher, 33.3 [31.8–35] μm min^−1^ and the catastrophe frequency is 0.16 [0.14–0.19] event min^−1^ (Fig. 1e–i). Those values are in great agreement with previous experiments from the lab and others [14]. The addition of EB1 from mice slowed down the depolymerization rate to 25.4 [22.2–25.4] μm min^−1^ (p-value < 0.001) but speeds up the growth rate to 1.7 [1.6–1.72] μm min^−1^ (p-value < 0.001) and increases the catastrophe frequency by around threefold to 0.50 [0.46–0.57] event min^−1^ (Fig. 1e–i). This observation corresponds to the well-known effect of mammalian EB1 on MTs dynamics described previously in reconstituted cell-free systems [15]. Surprisingly, in presence of EB1 from plant, MTs growth and shrinkage rates are decreased to 0.8 [0.78–0.83] μm min^−1^ (p-value < 0.001) and to 25.4 [25.4–27] μm min^−1^ (p-value < 0.001) respectively (Fig. 1e, g). Additionally, EB1 from plant has no effect on the catastrophe frequency, 0.17 [0.16–0.20] event min^−1^ (p = 0.364) (Fig. 1i, j). The rescue frequency couldn’t be compared because no rescue events were observed in any of the experiments carried out. Additionally, we decided to compute the dynamicity value for each condition in order to estimate and compare the rate of tubulin dimers exchange at the MT tip. The dynamicity is calculated as described in Vasquez et al. 1997 [16] and expressed in dimers sec^−1^. The comparison of the dynamicity reveals the opposite intrinsic properties that the mammalian and the plant protein have on MT dynamics. Indeed, the mammalian EB1 increases dynamicity from 57 dimers sec^−1^ in the control condition to 76 dimers sec^−1^ while the plant EB1 decreases it to 39 dimers sec^−1^.

## Conclusions

Our experiment suggests that the plant and the mammalian EB1 proteins have different intrinsic properties on MTs dynamics. Indeed, the mammalian EB1 is known to make microtubule more dynamic, increasing growth rate and catastrophe frequency while surprisingly the plant EB1-b protein seems to stabilize microtubules by reducing both growth and shrinkage rates. The opposite effects of EB1 from mice and EB1-b from plant on microtubule dynamics are well recapitulated by their effects on the dynamicity parameter (see Table 1 for details).

**Table 1:** Description of the parameters of microtubules dynamic instability for the three conditions used in this study

	Tub (15 µM)	Tub (15 µM) + EB1 mouse	Tub (15 µM) + AtEB1-b
N (MT)	66	41	65
N (Growth)	533	533	652
N (Shrinkage)	277	515	462
Growth time (sec)	105,375	60,965	152,680
Shrinkage time (sec)	3775	4165	4805
Growth distance (µm)	1951	1689	2054
Shrinkage distance (µm)	1948	1695	2047
Growth Rate (μm/min)	1.11	1.66	0.81
Shrinkage rate (μm/min)	30.96	24.42	25.56
Dynamicity (dimer/sec)	~ 58	~ 78	~ 41

Parameters have been pooled from all the available observations

### Limitations

In this work, we decided to purify and use EB1-b, among the three orthologs identified in *Arabidopsis,* because it is the one showing the strongest phenotypes in plants when knocked-out or mutated [11, 13] and the most similar one to its mammalian counterpart [4, 8, 9]. Knowing the dissimilarity in sequence between EB1-b and EB1-a or EB1-c, continuing this work by comparing EB1-a, EB1-b and EB1-c to the mammalian EB1 would give more information regarding the conservation of the intrinsic functions of those proteins. Furthermore, EB1 binds MT as a dimer and it seems that EB1-a and EB1-b can form homo- and hetero-dimers in vitro [17]. One could assess if the composition of the dimers changes their effect on MT dynamics. Moreover, in mammals and yeast EB1 is known to recruit a network of proteins to the microtubule tip. The effect of the combination of all these partners on microtubule dynamics is not known and could be more important than the intrinsic properties of EB1 itself. One could assess the ability of the mice and plant version of EB1 to recruit various mammalian and plant partners such as CLIP-170 or CLASP. Additionally, the experiment was done using purified tubulin from calf brain. Tubulin from different sources could influence the functions of EB1 from plant and mice due to differences in tubulin composition and/or post-translational modifications. Indeed, *Arabidopsis* presents a great diversity of alpha and beta tubulins and tubulin purified from tobacco possesses a different combination of post-translational modifications than brain tubulin [18]. Although, interpretation was made easier having a reference point (mammalian EB1 on brain tubulin) to which we could compare the plant EB1, reproducing the work using plant tubulin would be beneficial. Comparing the effect of mammalian and plant EB1 protein on the dynamics of MT formed with tubulin from brain or plant would tell us about the specificity of the MT-EB1 interaction for EB1 functions. EB1 is a plus-tip tracking protein in vivo and this activity can be reproduced in vitro but it is sensitive to both salt and EB1 concentration. Here, we used conditions known to allow the mammalian EB1 protein to tip-track and modulate MT dynamics. We assumed that the plant EB1 would behave the same way. One could reproduce the experiments with a fluorescently labelled plant EB1 to assess its ability to track MT tip (as opposed to lattice binding) and to compare comet length. Additionally, the MT network in *Arabidopsis* exhibits MT treadmilling [19], a process during which a MT simultaneously shrinks from its minus-end and grows from its plus-end. This phenomenon is rare in other eukaryotes. To reach such a state, the rates at both ends must be very similar. The ability of the plant EB1 proteins to dampen microtubule dynamics could be important in this context. Indeed, the rates at the minus-end being notoriously slow reducing the dynamic of the plus-end could help matching the dynamics of the two ends. This possible role could be evaluated by measuring the proportions of treadmilling MTs in plant lacking EB1 proteins.

## Supplementary information

**Additional file 1.** Additional methods on protein expression, purification, and sample preparation.

## Data Availability

The datasets analyzed during the current study are available from the corresponding authors.

## Abbreviations

MAPs: Microtubule Associated proteins
EB1: End-binding protein 1
MT: Microtubule
