# Supplementary material for: Plant and mouse EB1 proteins have opposite intrinsic properties on the dynamic instability of microtubules
Source: BMC Res Notes. 2020 Jun 22;13:296. doi: 10.1186/s13104-020-05139-6 (PMC7310003; doi:10.1186/s13104-020-05139-6)
Supplement: Supplementary file 1 — Additional file 1. Additional methods on protein expression, purification, and sample preparation. [file 13104_2020_5139_MOESM1_ESM.docx]

**Molines *et al.*, 2020, Supplemental Material**

**Methods**

Protein expression and purification

*EB1* – Histidine-tag recombinant mouse EB1 was purified as described previously [1] and stored in a BRB80 buffer (80 mM 1,4-piperazinediethanesulfonic acid, 1 mM ethylene glycol tetraacetic acid, 1 mM MgCl2, pH 6.8) containing 50 mM KCl.

*EB1-b* – The pET32b plasmid containing the EB1-b sequence from Komaki *et al.* 2010 [2] was transformed in *E. coli* BL21 pLys S strain. Bacteria were grown at 37°C overnight, diluted back to 0.05 OD_600_ and then grew at 37°C until OD_600_ reached 0.5. At that point, expression was induced with IPTG at 1 mM for 5h at 32°C. After centrifugation, the pellet was suspended in 15 mL of lysis buffer then placed on ice for 30 minutes. The lysis buffer contains TRIS at 40 mM (pH 7 with HCl), NaCl at 300 mM, Triton X-100 at 0.1%, lysozyme at 1 mg.mL^-1^, aprotinin at 10 μg.mL^-1^, leupeptin at 10 μg.mL^-1^, pepstatin A at 1 μg.mL^-1^ and PMSF at 1 mM. Cells were lysed by sonication. 2000 units of DNAse II were added to the solution which was left on ice for 10 minutes. The solution was centrifuged at 25.000 *g* at 4°C for 30 minutes. After equilibration of a Talon cobalt high affinity resin (Takara Bio) with 20 mM TRIS pH 7, NaCl at 300 mM, Triton X-100 at 1% (v/v) and imidazole at 10 mM, the resin is incubated with the supernatant for 40 minutes at 4°C. The flow through is removed by centrifugation at 4°C at 700 *g* for 5 minutes. The resin is washed three times with the equilibration buffer. Each wash lasts 15 minutes at 4°C. The protein is then eluted by fraction of 500 μL, at 4°C with a buffer of TRIS at 20 mM (pH 7 with HCl), NaCl at 150 mM and imidazole at 200 mM. The resulting purified protein was processed further through exclusion chromatography (Superdex 200 gel filtration column—GE-Healthcare, 17-5175-01) to ensure purity and eluted in BRB80 buffer.

*Tubulin* - Tubulin was purified from calf brains [3] and fluorescently labelled with ATTO-488 or ATTO-565 fluorophores, as previously described [4].

*Protein concentration* – Protein concentration (for both tubulin and EB1s) was estimated using the Bradford method.

Sample preparation

A perfusion chamber was made from cleaned glass slide and a coverslip using double sided tape. Slide and coverslip were cleaned as described in Portran et al., 2013 [5]. The chamber was perfused with Neutravidine (25 μg.mL-1 in BRB80/BSA 1% (m/v)) for 2 minutes, PLL-PEG for 30 sec (2 kDa 0.1 mg.ml-1 in 10mM Hepes, pH 7.4), BRB80/BSA 1%, GMPCPP-stabilized, ATTO-565-labeled microtubule seeds [6] were then perfused for 5 minutes before washing the chamber three times with BRB80/BSA 1% and then flowing the reaction mix. The reaction mix contained BRB80, complemented with 1% BSA, 50 mM KCl, 1 mg.mL-1 glucose , 70 μg.ml-1 of catalase, 580 μg.ml-1 glucose oxidase, 4 mM DTT, 1 mM GTP, 0.017% methyl-cellulose (m/v), 10 μM of unlabeled tubulin and, 5 μM ATTO-488-labeled tubulin. After the perfusion of the reaction mix, the chamber was sealed with putty and then placed on the microscope stage at 36°C for imaging.

**References**

1. Vitre B, Coquelle FM, Heichette C, Garnier C, Chrétien D, Arnal I. EB1 regulates microtubule dynamics and tubulin sheet closure in vitro. Nat Cell Biol. 2008;10:415–21.

2. Komaki S, Abe T, Coutuer S, Inzé D, Russinova E, Hashimoto T. Nuclear-localized subtype of end-binding 1 protein regulates spindle organization in Arabidopsis. J Cell Sci. 2010;123:451–9.

3. Gell C, Friel CT, Borgonovo B, Drechsel DN, Hyman AA, Howard J. Purification of tubulin from porcine brain. Methods Mol Biol. 2011;777:15–28.

4. Hyman AA. Preparation of marked microtubules for the assay of the polarity of microtubule-based motors by fluorescence. In: Journal of Cell Science. 1991. p. 125–7.

5. Portran D, Zoccoler M, Stoppin-MelletGaillard J, Stoppin-Mellet V, Neumann E, Arnal I, et al. MAP65/Ase1 promote microtubule flexibility. Mol Biol Cell. 2013;24:1964–73.

6. Ramirez-Rios S, Serre L, Stoppin-Mellet V, Prezel E, Vinit A, Courriol E, et al. A TIRF microscopy assay to decode how tau regulates EB’s tracking at microtubule ends. In: Methods in Cell Biology. 2017. p. 179–97.

| 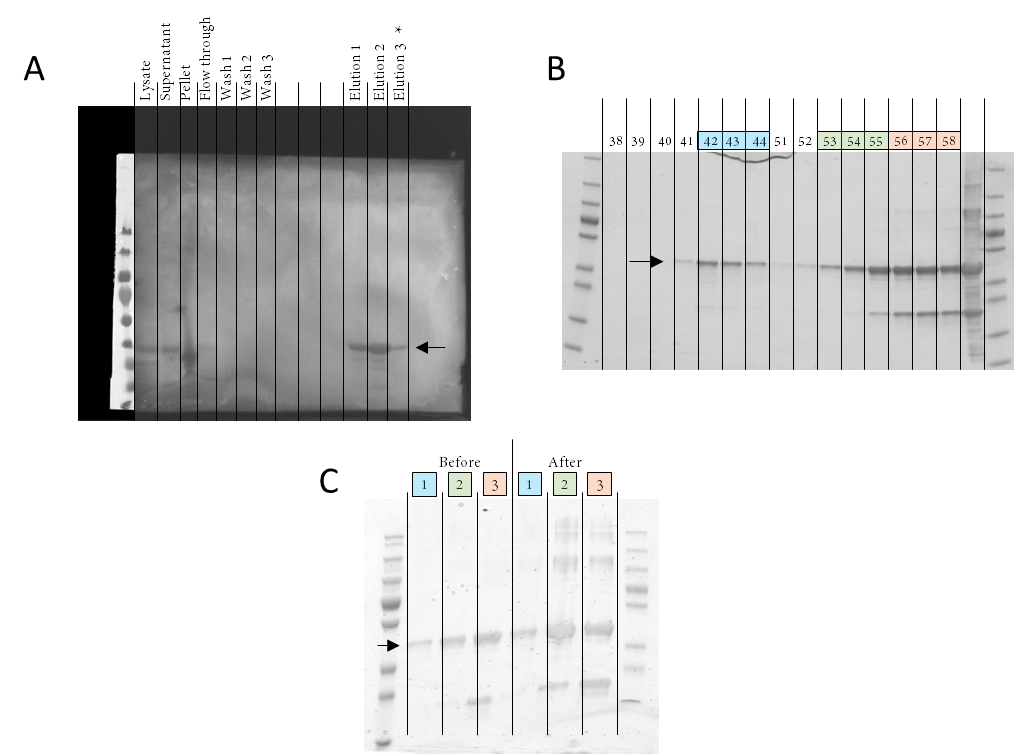 |
| --- |
| **Sup. Figure 1: Procedure for the purification of plant EB1b protein.**  **(A)** Purifications by affinity chromatography (His-Tag). Each step of the purification was tested by SDS PAGE electrophoresis using 20 µL samples of, from left to right respectively, (1) bacterial lysate, (2) supernatant and (3) pellet after centrifugation of the lysate to remove insoluble elements, (4) flow through after the incubation step with TALON beads, (5-7) three successive washes and the (11-13) 3 elution fractions of interest (identified by a rapid Bradford's test). **(B)** Subsequent purification by exclusion chromatography using 200 µL of the purified protein. The numbers correspond to the elution fractions collected sequentially. Fractions #42 to #44 were pooled into pool 1, #53 to #55 into pool 2 and #56 to #59 into pool 3. Pool 1 was used for the in vitro experiments. **(C)** Comparison of the protein quality before and after the gel filtration. The band corresponding to the EB1b protein is indicated with a black arrow. The numbers above the tracks correspond to the fraction pools described before. |
